# RETRACTION:  How blockchain-timestamped protocols could improve the trustworthiness of medical science

**DOI:** 10.12688/f1000research.11888.1

**Published:** 2017-06-08

**Authors:** Greg Irving, John Holden

**Affiliations:** 1 Institute of Public Health, University of Cambridge, Cambridge, CB2 OSR, UK; 2 General Practitioner, Garswood Surgery, St. Helens, Lancashire, WN4 0XD, UK

At the request of the authors Greg Irving and John Holden, the article titled “How blockchain-timestamped protocols could improve the trustworthiness of medical science” has been retracted from F1000Research. The authors have taken this decision after considering the methodological concerns raised by a peer reviewer during the post-publication open peer review process. As the methodology has been deemed to be unreliable, the article is now retracted. This applies to all three versions of the article: Irving G and Holden J. How blockchain-timestamped protocols could improve the trustworthiness of medical science [version 1; referees: 2 approved]. F1000Research 2016, 5:222 (doi: 10.12688/f1000research.8114.1) Irving G and Holden J. How blockchain-timestamped protocols could improve the trustworthiness of medical science [version 2; referees: 3 approved]. F1000Research 2016, 5:222 (doi: 10.12688/f1000research.8114.2) Irving G and Holden J. How blockchain-timestamped protocols could improve the trustworthiness of medical science [version 3; referees: 3 approved, 1 not approved]. F1000Research 2017, 5:222 (doi: 10.12688/f1000research.8114.3).

